# Weekly Acupuncture for a Patient With Hemifacial Spasms: A Case Report

**DOI:** 10.7759/cureus.55219

**Published:** 2024-02-29

**Authors:** Go Horibe, Satoru Yamaguchi, Ai Kouchi, Shintaro Ibata, Toshimasa Yamamoto

**Affiliations:** 1 Department of Oriental Medicine, Saitama Medical University, Saitama, JPN; 2 Department of Oriental Medicine, Saitama Medical University Kawagoe Clinic, Saitama, JPN; 3 Department of Neurology, Saitama Medical University, Saitama, JPN

**Keywords:** clinical case report, facial nerve, electroacupuncture, acupuncture, hemifacial spasm

## Abstract

The efficacy and optimal frequency of acupuncture for hemifacial spasms (HFSs) in patients unresponsive or averse to standard treatment methods remains unestablished. Here, we administered acupuncture to a patient with HFSs who was dissatisfied with the outcomes of botulinum toxin (BoNT) injections as symptomatic treatment. A man in his 60s, experiencing frequent spasms in his left facial muscles since 2015, had received several BoNT injections without receiving microvascular decompression or medication; however, the treatment results were not satisfactory. In 2020, he visited our clinic for acupuncture. His entire face twitched involuntarily, and the other Babinski sign was observed. The spasm severity was 5 on the numerical rating scale (NRS). Acupuncture was performed on the gallbladder meridian (GB) 2, stomach meridian (ST) 7, and triple energizer meridian(TE) 17 along the facial nerve and GB14, GB1, small intestine meridian (SI) 18, ST4, ST5, and ST9 on the affected (left) side. In the fourth session, 1 Hz electroacupuncture at ST7 and TE17 reduced the NRS score to 1. As his spasms were well managed, we initially continued with biweekly acupuncture sessions. However, by the 10th session, a worsening of symptoms led to a revert to weekly treatment, which maintained a decreased NRS score until the 21st session. Our findings suggest that weekly acupuncture may be a viable treatment modality for patients with HFSs unresponsive or averse to conventional treatments. Future prospective clinical trials are required to verify the efficacy of acupuncture for HFSs.

## Introduction

Hemifacial spasms (HFSs) are characterized by involuntary contractions or spasms in the facial muscles on one side of the face. The incidence of HFSs is reported to be 0.78 per 100,000 individuals [1]. Almost all patients with HFSs experience these spasms due to demyelination and false synapse formation (ephapses) or facial nerve compression by the anterior, posterior cerebellar, or vertebral artery [1]. An alternative hypothesis suggests that abnormal retrograde conduction due to facial nerve compression by blood vessels leads to hyperfunction of the facial nerve nucleus and subsequent facial spasms [2].

The standard treatments for HFSs include microvascular decompression (MVD), botulinum toxin (BoNT) injection, and other medications. In 1975, Jannetta et al. reported MVD as a method for alleviating facial muscle spasms via separating the blood vessels from the affected facial nerve [3]. Later, a systematic review of 39 studies reported a spasm-free rate of 90.5% in patients with HFSs after MVD treatment [4]. BoNT injections are frequently used as a conservative treatment option for HFSs, with efficacy ranging from 73% to 98.4% [5]. However, high-quality randomized controlled trials (RCTs) evaluating BoNT injection benefits have not been conducted. In addition, some antiepileptic drugs have been used to treat HFSs [6]. Meanwhile, some patients with HFSs utilize complementary and alternative medicine, such as acupuncture, facial massage, and health supplements [7].

Acupuncture, a traditional East Asian medical treatment modality, lacks demonstrated effectiveness in treating patients with HFS, mainly owing to a lack of high-quality research studies and reliable data. A systematic review of acupuncture studies for HFS treatment deemed the studies to be of low quality, raising doubts about their effectiveness [8]. Thus, elucidating the effectiveness and efficacy of acupuncture for treating patients with HFS is essential.

This case report describes the use of weekly acupuncture to treat subjective spasms in a patient with HFSs who did not experience satisfactory improvement despite receiving BoNT injections and had not been treated with MVD or other medications. A significant improvement in subjective spasms was observed following acupuncture. The patient was informed about the acupuncture methods, and informed consent was obtained for data publication.

This article was previously presented as a meeting abstract at the 40th Congress of the Japanese Society of Neurological Therapeutics in November 2022.

## Case presentation

The patient was a male in his 60s who first noticed spasms in his orbicularis oculi muscle around 2015, gradually progressing to the entire face. In 2018, he sought consultation with the Department of Neurology at Saitama Medical University Hospital, Japan. Earlier tibial fracture treatment with a metal plate precluded a brain MRI, leading to a plain computed tomography (CT) of the cerebrum to rule out macroscopic lesions. Clinical symptoms suggested HFS; however, vascular compression of the facial nerve could not be confirmed, and MVD was not performed since MRI was not feasible. Despite multiple BoNT injections, the patient did not perceive any improvement in his spasms and discontinued treatment. In October 2020, the patient visited our clinic, hoping to improve his condition. The presentation included a history of hypertension, overactive bladder, and various medications, including losartan potassium, nifedipine, solifenacin succinate, epinastine hydrochloride, and vonoprazan fumarate. Brotizolam was taken as needed for insomnia, but no antiepileptic drugs were used. During our initial clinic visit, his height measured 165 cm, and he weighed 64 kg. His entire face displayed involuntary twitching, and the other Babinski signs were observed. No facial muscle paralysis or other neurological abnormalities were observed. Consistent with previous physicians, we diagnosed his condition as HFS.

Acupuncture methods

**Figure 1:**
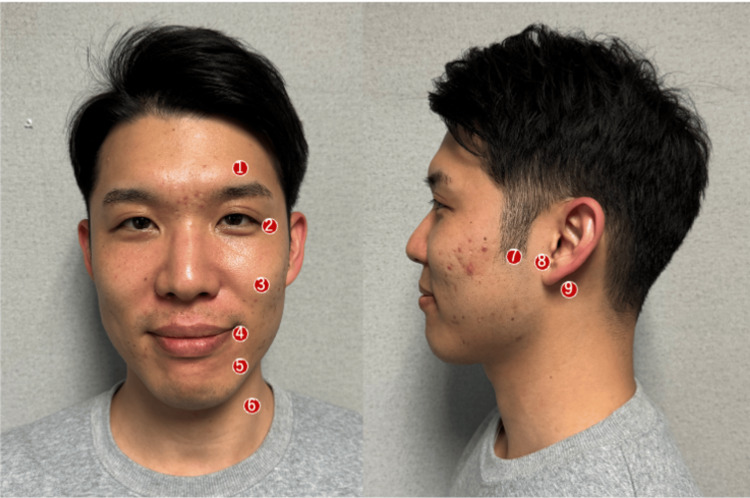
Acupuncture site 1) Gallbladder meridian (GB) 14, 2) GB1, 3) small intestine meridian (SI) 18, 4) stomach meridian (ST) 4, 5) ST5, 6) ST9, 7) ST7, 8) GB2, 9) triple energizer meridian (TE) 17. The person in the photo is the author (GH) and not the patient in this case.

Acupuncture was administered to enhance facial nerve function. We inserted 10-mm needles at the gallbladder meridian (GB) 2, stomach meridian (ST) 7, and triple energizer meridian (TE) 17 along the facial nerve and at the GB14, GB1, small intestine meridian (SI) 18, ST4, ST5, and ST9 on the facial muscles (Figure 1). Acupuncture needles were inserted vertically into the skin for GB2, ST7, and TE17 points and horizontally into the other acupuncture points on the facial muscles. After confirming the qi sensation, the needles were left in place without further manipulation. Disposable needles (Seirin Co., Shizuoka. Japan, length: 40 mm, diameter: 0.16 mm) were used for the procedure. From the fourth acupuncture session onward, in addition to the previous acupuncture, we performed electroacupuncture with a frequency of 1 Hz and amplitude of 200 μs at ST7 and TE 17. Acupuncture was performed 21 times, with treatment frequency varying between once a week to once every two weeks; each session lasted 30 minutes. Only one acupuncturist with nine years of experience was involved in the treatment of this case.

Outcome measurement

The outcome was assessed using the numeric rating scale (NRS) to evaluate the degree of spasms experienced by the patients. The NRS score ranges from 0 (no seizures) to 10 (highest possible spasm intensity) and was evaluated by an acupuncturist during treatment.

NRS timecourse

**Figure 2:**
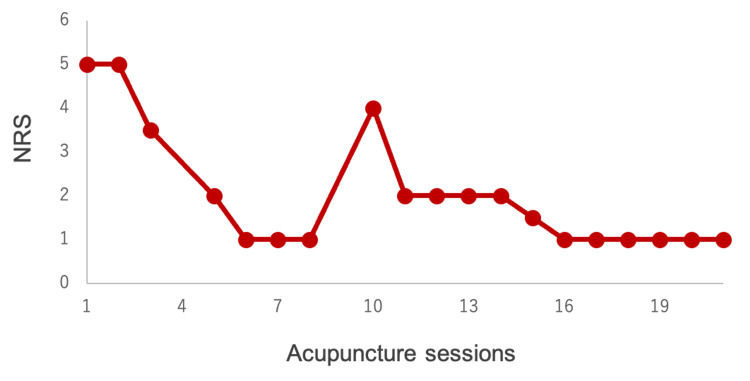
Course of the numerical rating scale (NRS) to monitor the degree of spasms.

The progression of NRS scores is shown in Figure 2, with a baseline NRS of 5. To further improve his spasms during the fourth visit for treatment, we performed electroacupuncture at ST7 and TE 17, after which the NRS score decreased to 1. The patient reported reduced severity and frequency of HFS with EA. As the spasms were well managed, acupuncture frequency was changed from once a week to once every two weeks for treatment sessions 6 through 9. However, when he visited us after two weeks for his tenth session, his spasms had worsened. 

Consequently, we reverted to the frequency of once per week for subsequent sessions and monitored the progression of symptoms. This led to an NRS score decrease to 1, which remained unchanged until the 21st treatment session. No serious adverse events occurred throughout the treatment period, although a small amount of bleeding occurred infrequently following needle removal.

Patient perspective

During the initial visit, the patient noted spasms characterized by synchronous contractions of the muscles of his entire face. After initiating acupuncture, the patient reported improved facial symmetry at his second visit. At the fifth visit, he stated that the spasm from his cheeks to his anterior neck was remarkably reduced. After the sixth visit, acupuncture frequency was reduced to once every two weeks. At the 10th visit, he stated that the spasms were still persistent despite the acupuncture; thus, we reverted to weekly acupuncture, and by the 15th visit, he noted that the frequency of his facial muscle spasms had decreased.

## Discussion

Our findings suggest that HFS symptoms in patients not undergoing standard therapy may be alleviated by EA. In this case, a brain MRI could not be performed due to a metal plate implanted into the tibia. Consequently, MVD was not possible as it was unclear whether the facial nerve was compressed by cerebral vessels. As an asymptomatic therapy, the patient received several BoNT injections but remained dissatisfied with the outcomes. Furthermore, alternative pharmacological interventions to alleviate the spasms were not explored. However, following acupuncture, the patient’s self-reported symptoms were alleviated, and his suffering was significantly reduced.

The actual efficacy of acupuncture for HFSs remains inconclusive. A systematic review reported higher total efficacy and clinical cure rates than a control group. However, bias was not examined, and the quality and accuracy of the studies included in the analysis were unclear [8]. An overarching report on systematic reviews of acupuncture for patients with HFSs published in 2022 stated that acupuncture was more effective than Western medicine in treating HFSs. However, when these reviews were evaluated using AMSTAR-2, PRISMA, ROBIS, and GRADE guidelines, the overall quality of the systematic reviews was low [9]. Therefore, the true efficacy of acupuncture for patients with HFSs remains unclear. In another case study of acupuncture efficacy in HFS patients with temporomandibular joint disorder, a combination of EA and manual acupuncture decreased the R2 of the blink reflex [10]. This report indicates that EA conducted at ST7 and SI18 along the facial nerve pathway, as performed in the present study, may improve facial nerve functions. In addition, a case series by Yamamoto and Nishimura reported that transcutaneous electrical nerve stimulation (TENS) along the facial nerve reduced facial nerve spasm frequency and severity in several patients with HFS [11].

Other studies have evaluated the therapeutic effects of acupuncture on the activity of motor nerves other than the facial nerves. In a study that examined whether acupuncture could suppress heightened somatic motor neuron activity elicited by the vibration-induced finger flexion reflex, acupuncture stimulation at LI4 suppressed finger flexion and integrated surface EMG [12]. Moreover, needle stimulation can suppress the tonic vibration reflex [13]. These reports suggest that acupuncture to the face may have affected facial nerve function in our patient, resulting in a reduced severity of facial muscle spasms. Based on these reports, it is possible that EA at acupuncture points along the facial nerve in our patient suppressed facial nerve function and improved spasms. Nevertheless, the inhibitory responses observed in motor neurons are transient. In our case, the reduction in the NRS score due to continued acupuncture suggests that the inhibitory response of motor neuron activity may be only part of the mechanism of action of acupuncture, and other currently unknown factors may also contribute to its effectiveness.

The frequency of acupuncture is often a cause of concern. Studies have assessed the effective number of acupuncture sessions for conditions, such as migraines [14], chronic pelvic pain syndrome [15], stroke [16], and tinnitus [17]. In Japan, acupuncture is traditionally performed once a week, and our clinic often follows this schedule. However, acupuncture treatment is not generally covered by the Universal Health Insurance System in Japan, placing a substantial economic burden on the patient. In the present case, the facial spasms of the patient had improved. Considering the economic burden on this patient, the frequency of acupuncture treatment was changed to once every two weeks, but the patient’s symptoms worsened. This further supports the traditional wisdom that acupuncture should be administered at least once a week for effective alleviation of unilateral facial spasms.

This case report had several limitations. First, the inability to perform brain MRI and the absence of digital subtraction angiography and CT angiography were due to the clinician's decision at the time. As a result, it was unclear whether the facial nerve was compressed by a cerebral blood vessel. Differential diagnoses of HFS include blepharospasm and Meige's syndrome, but we suspect that this case does not fall into either category because of the clinical characteristics of the patient's presentation. Blepharospasm, a disorder similar to HFS but with unilateral spasms, followed quickly by bilateral spread. In addition, the spasm is usually confined to the eyelid [18]. Meige's syndrome begins with eyelid spasms that gradually follow the contralateral eyelid and spread to the face and neck outside the eyelid, so again, bilateral symptoms are common [19]. Since our patient had unilateral spasms of the entire face accompanied by the other Babinski sign, we consider it highly likely that our patient had HFS. Nevertheless, HFS was inferred from the clinical symptoms alone, and the pathophysiology remained uncertain. The second limitation was using the NRS to measure the degree of spasms. The NRS is a simple system frequently used in clinical settings; however, it may contain significant bias as it is based on subjective evaluation. Objective evaluations, such as video recordings or electromyograms, may provide more reliable results. Unfortunately, our outpatient booth does not have such facilities.

## Conclusions

Acupuncture is a potential treatment option for patients who are unresponsive to or have declined conventional therapies. Moreover, although our results provide evidence that weekly acupuncture sessions may be beneficial for treating HFS, it is advisable to exercise caution regarding the frequency of acupuncture until more substantial data is gathered. Thus, it is essential to conduct prospective clinical studies in the future.
